# Supplementary figures and images for: High-throughput FastCloning technology: A low-cost method for parallel cloning
Source: PLoS One. 2022 Sep 9;17(9):e0273873. doi: 10.1371/journal.pone.0273873 (PMC9462701; doi:10.1371/journal.pone.0273873)

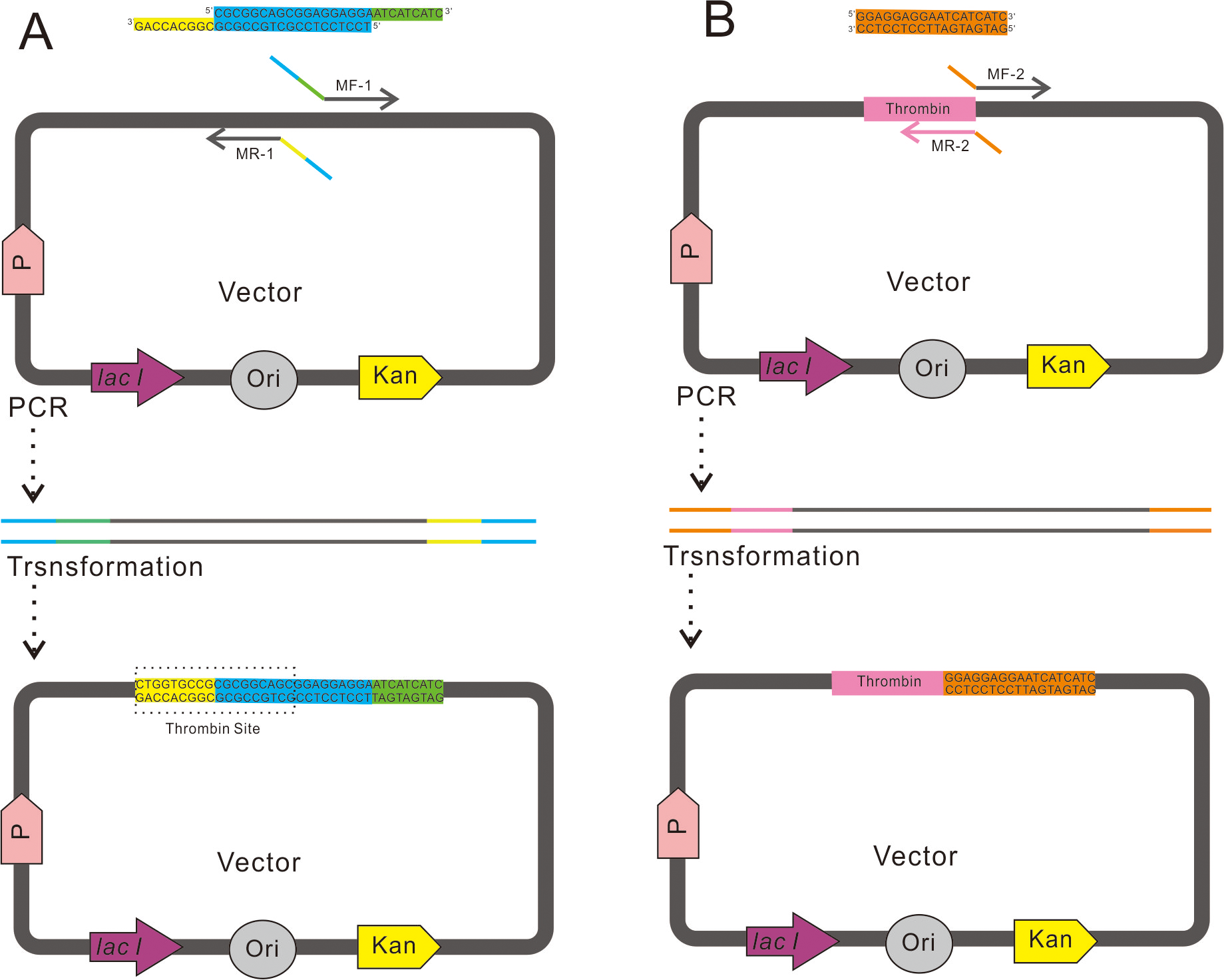

Supplement: S1 Fig — (TIF) [file pone.0273873.s001.tif]

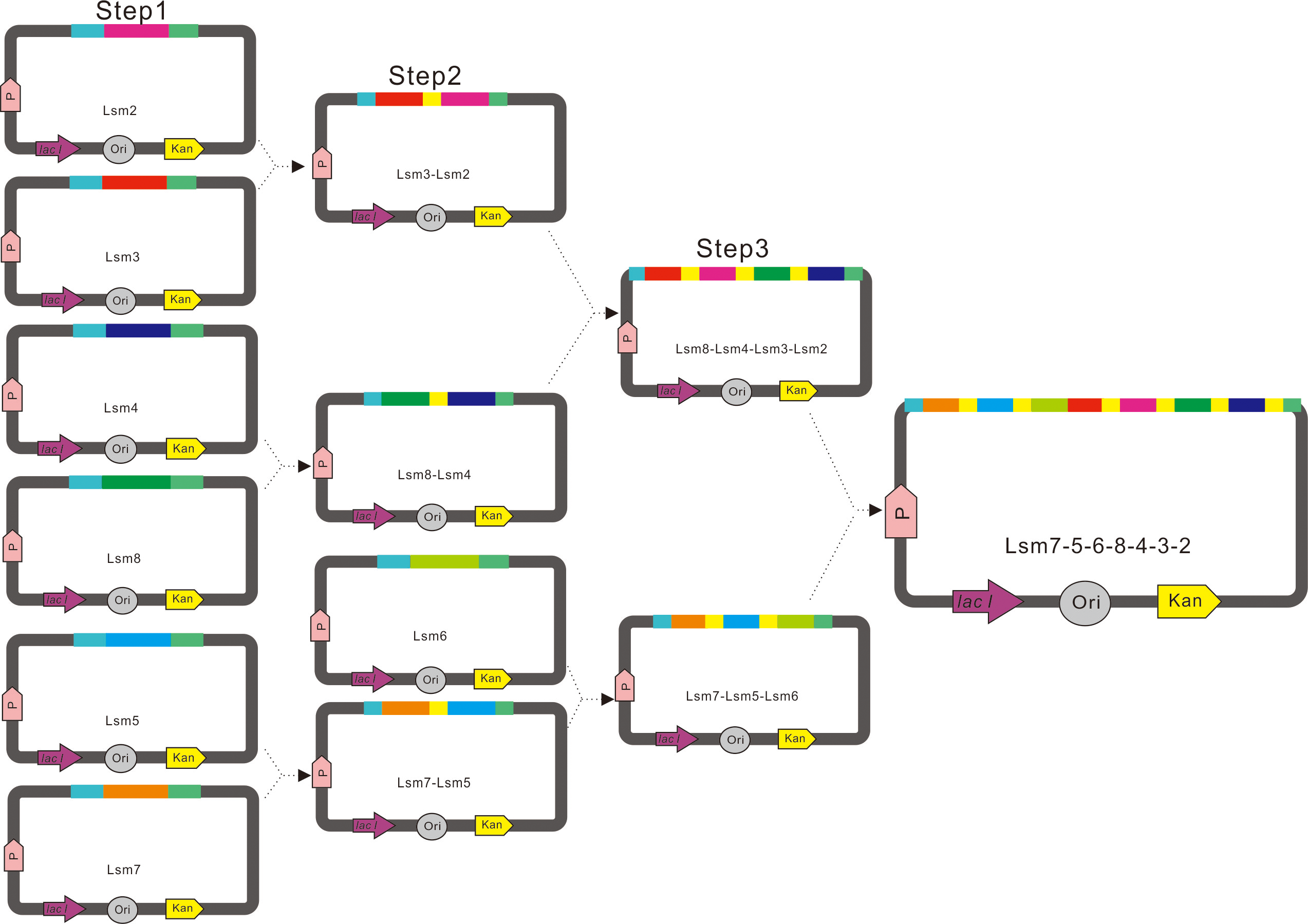

Supplement: S2 Fig — (TIF) [file pone.0273873.s002.tif]

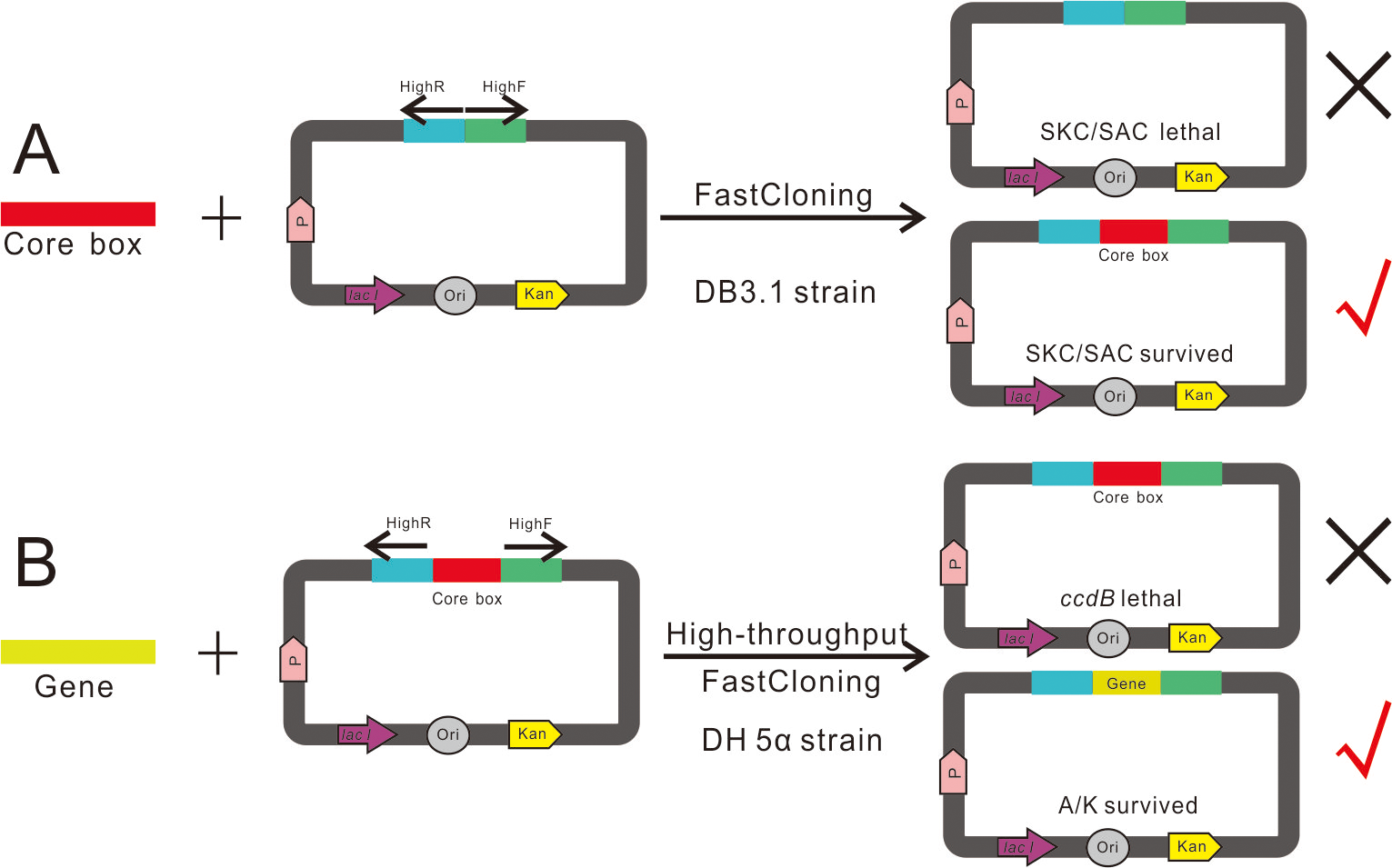

Supplement: S3 Fig — (TIF) [file pone.0273873.s003.tif]

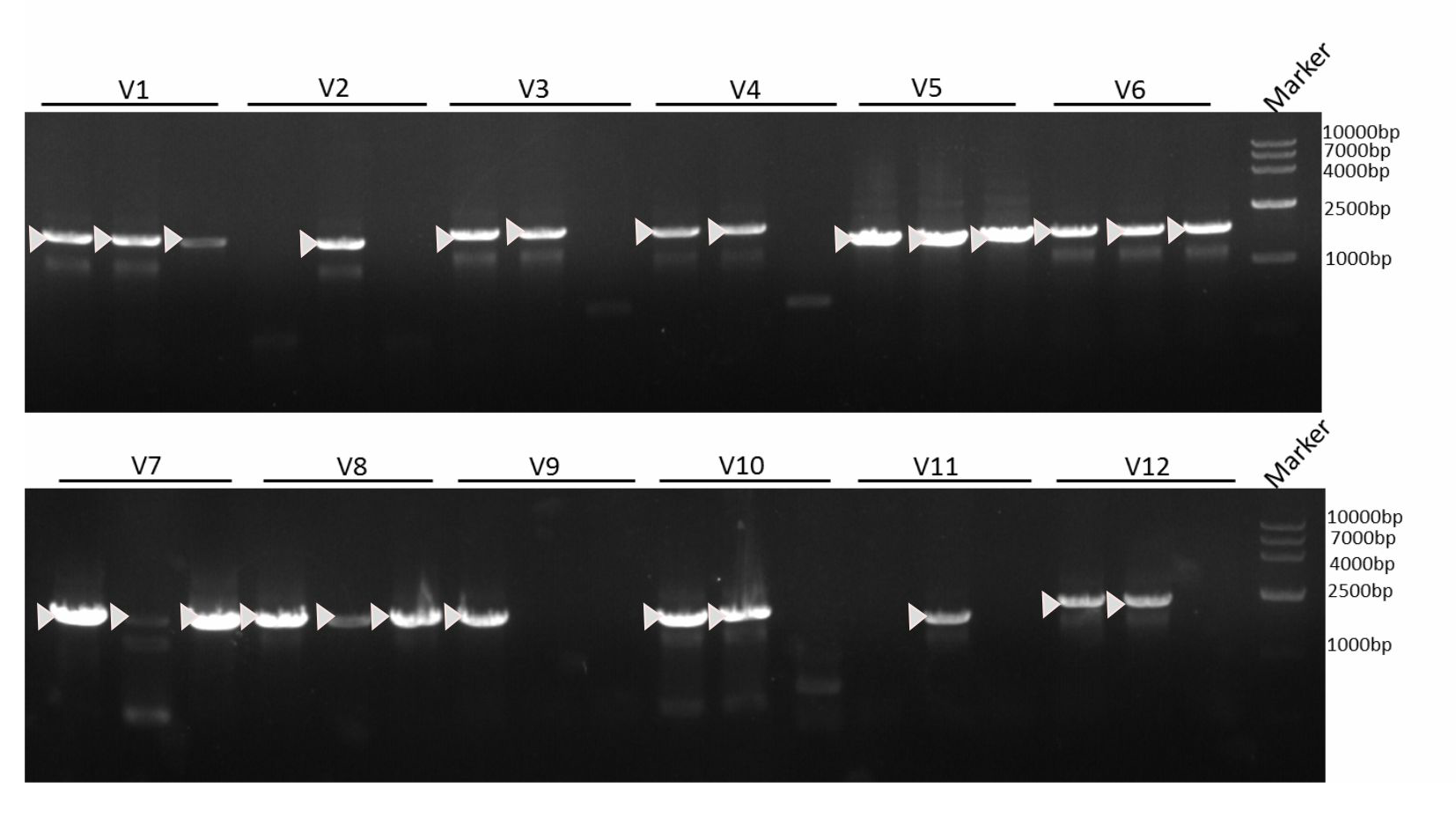

Supplement: S4 Fig — (TIF) [file pone.0273873.s004.tif]

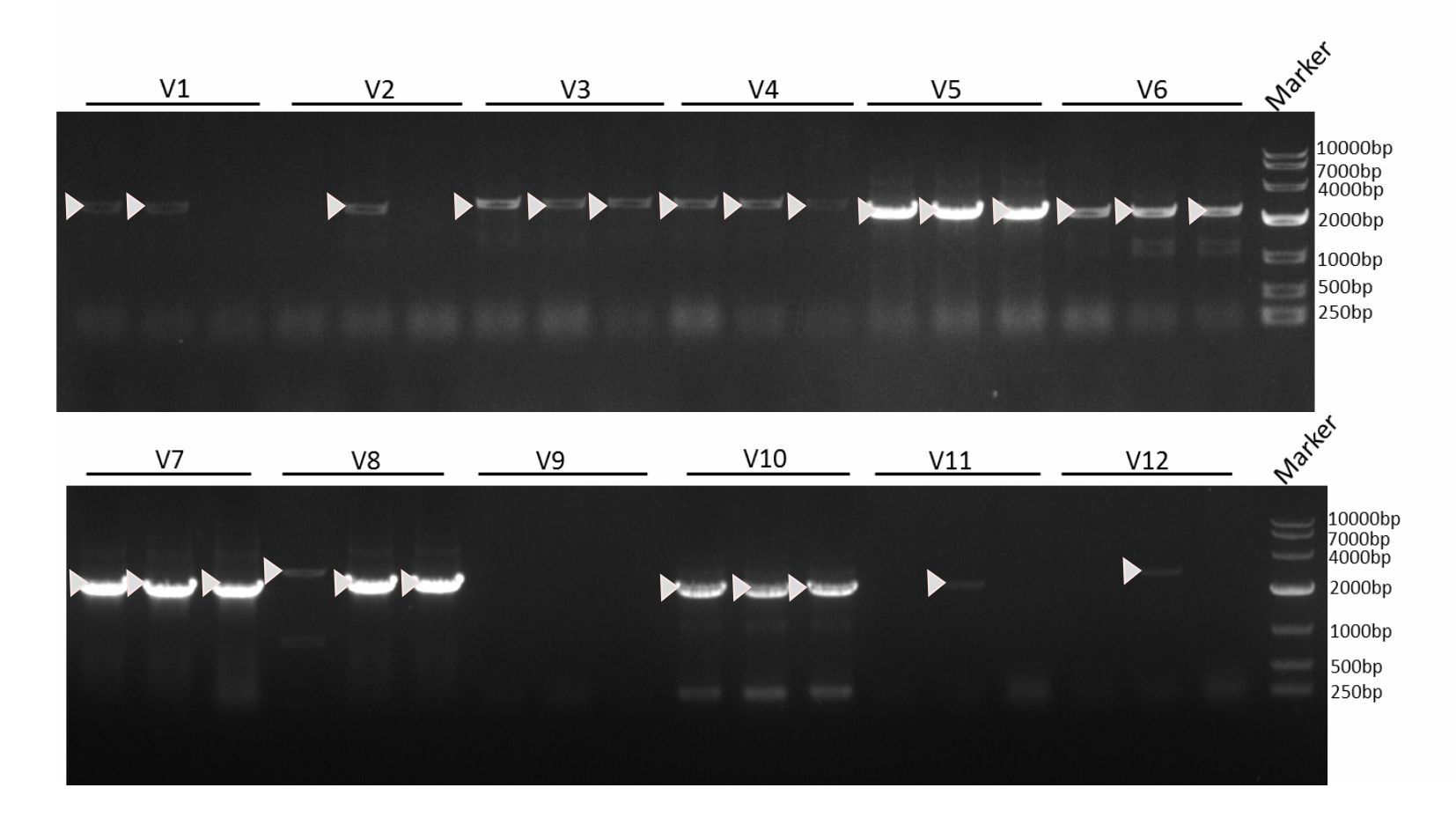

Supplement: S5 Fig — (TIF) [file pone.0273873.s005.tif]

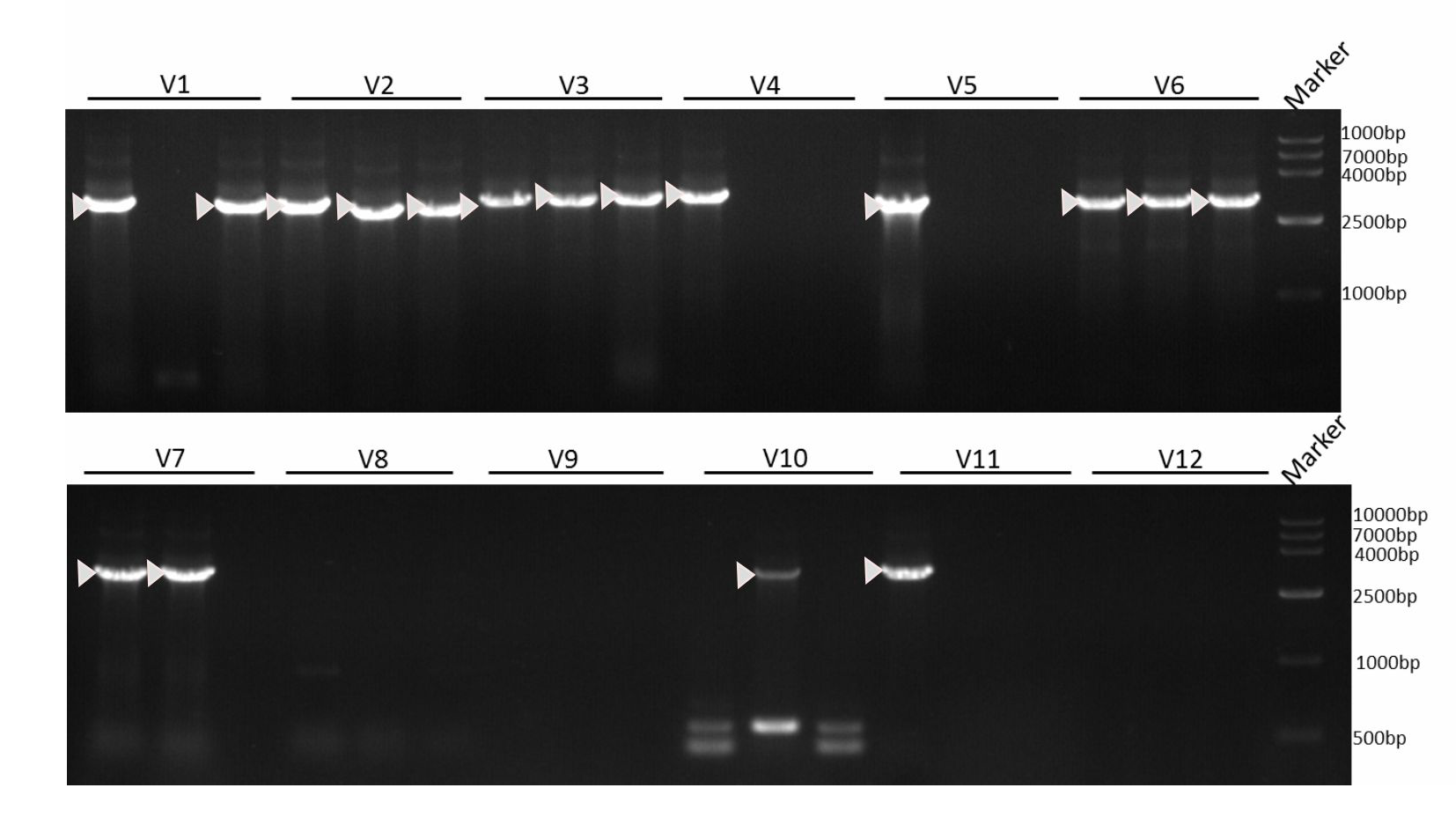

Supplement: S6 Fig — (TIF) [file pone.0273873.s006.tif]
