# Supplementary material for: High-throughput FastCloning technology: A low-cost method for parallel cloning
Source: PLoS One. 2022 Sep 9;17(9):e0273873. doi: 10.1371/journal.pone.0273873 (PMC9462701; doi:10.1371/journal.pone.0273873)
Supplement: S1 Table — (DOCX) [file pone.0273873.s007.docx]

S1 Table. The information of second generation high-throughput prokaryotic expression vectors.

| **Vector** | **Host** | **Affinity tag** | **Tag location** | | **Notes** |  |
| --- | --- | --- | --- | --- | --- | --- |
| pSDB1 | *E.coli* | 6×His | N |  | |  |
| pSDB2 | *E.coli* | 6×His | C |  | |  |
| pSDB3 | *E.coli* | 6×His-SUMO | N |  | |  |
| pSDB4 | *E.coli* | 6×His-SUMO | C |  | |  |
| pSDB5 | *E.coli* | 6×His-MBP | N |  | |  |
| pSDB6 | *E.coli* | 6×His-MBP | C |  | |  |
| pSDB7 | *E.coli* | GST | N |  | |  |
| pSDB8 | *E.coli* | GST | C |  | |  |
| pSDB9 | *E.coli* | 6×His | N | Two MCS sites | |  |
| pSDB10 | *E.coli* | 6×His | N | Cold-shock expression | | |
| pSDB11 | *E.coli* | 6×His | N |  | |  |
| pSDB12 | *E.coli* | 6×His | N |  | |  |
