# Supplementary material for: High-throughput FastCloning technology: A low-cost method for parallel cloning
Source: PLoS One. 2022 Sep 9;17(9):e0273873. doi: 10.1371/journal.pone.0273873 (PMC9462701; doi:10.1371/journal.pone.0273873)
Supplement: S2 Table — (DOCX) [file pone.0273873.s008.docx]

S2 Table. Primers used to modify high-throughput constructs.

| Name | Discription | Sequence |
| --- | --- | --- |
| MF1 | Insert 36 bp sequence to pFastV1 | GGAGGAGGAATCATCATCCATATGGCTAGCATGACTGGTGGAC |
| MR1 | Insert 36 bp sequence to pFastV1 | GATGATGATTCCTCCTCCGCTGCCGCGCGGCACCAGGCCGCTG |
| MF2-1 | Mutation AGC to TAA and delete intrinsic Thrombin site sequence to pFastV1 | TAAGAAAACCTGTATTTTCAGGGCATGGCTAGCATGACTGGTGGAC |
| MR2-1 | Mutation AGC to TAA and delete intrinsic Thrombin site sequence to pFastV1 | GCCCTGAAAATACAGGTTTTCTTAGTGATGATGATGATGATGGC |
| MF2-2 | Insert 36 bp sequence to pFastV2 | CGCGGCAGCGGAGGAGGAATCATCATCCATCATCATCATCATCACTAAG |
| MR2-2 | Insert 36 bp sequence to pFastV2 | TCCTCCTCCGCTGCCGCGCGGCACCAGGCTGCTGCCCATGGTATATCTCCTT |
| MF3-1 | Mutation pFastV3&4 intrinsic Thrombin site to TEV site | AACCTGTATTTTCAGGGCCATATGGCTAGCATGTCGGACTCAGAAGTC |
| MR3-1 | Mutation pFastV3&4 intrinsic Thrombin site to TEV site | CTGAAAATACAGGTTTTCGCCGCTGCTGTGATGATGATGATGATGGCT |
| MF3-2 | Insert 36 bp sequence to pFastV3 | CGCGGCAGCGGAGGAGGAATCATCATCTCCGAATTCGAGCTCCGTCGACAAG |
| MR3-2 | Insert 36 bp sequence to pFastV3 | TCCTCCTCCGCTGCCGCGCGGCACCAGTCCACCAATCTGTTCTCTGTGAGC |
| MF4 | Insert 36 bp sequence to pFastV4 | CGCGGCAGCGGAGGAGGAATCATCATCGAAAACCTGTATTTTCAGGGCCAT |
| MR4 | Insert 36 bp sequence to pFastV4 | TCCTCCTCCGCTGCCGCGCGGCACCAGGCCGCTGCTGTGATGATGATGATG |
| MF5-1 | Delete pFastV5 intrinsic Thrombin site | CATCATCACAGCAGCGGCATGAAAATCGAAGAAGGTAA |
| MR5-1 | Delete pFastV5 intrinsic Thrombin site | GCCGCTGCTGTGATGATGATGATGA |
| MF5-2 | Insert 36 bp sequence to pFastV5 | CGCGGCAGCGGAGGAGGAATCATCATCGATCCGGAATTCAAAGGCCTACGTC |
| MR5-2 | Insert 36 bp sequence to pFastV5 | TCCTCCTCCGCTGCCGCGCGGCACCAGCATGGCGCCCTGAAAATACAGGTTT |
| MF6-1 | Insert 36 bp sequence to pFastV6 | CGCGGCAGCGGAGGAGGAATCATCATCCATATGAAAATCGAAGAAGGT |
| MR6-1 | Insert 36 bp sequence to pFastV6 | TCCTCCTCCGCTGCCGCGCGGCACCAGGC |
| MF6-2 | Insert TEV site behind 36 bp sequence to pFastV6 | AACCTGTATTTTCAGGGCATGAAAATCGAAGAAGGTAAACTGGTAATC |
| MR6-2 | Insert TEV site behind 36 bp sequence to pFastV6 | CTGAAAATACAGGTTTTCGATGATGATTCCTCCTCCGCTGCCGCGCGG |
| MF7 | Insert 36 bp sequence to pFastV7 | CGCGGCAGCGGAGGAGGAATCATCATCGGATCCCCGGAATTCCCGGGTCGAC |
| MR7 | Insert 36 bp sequence to pFastV7 | TCCTCCTCCGCTGCCGCGCGGCACCAGGGGCCCCTGGAACAGAACTTCCAG |
| MF8-1 | Insert 36 bp sequence to pFastV8 | CGCGGCAGCGGAGGAGGAATCATCATCTCCCCTATACTAGGTTATTGGAAAAT |
| MR8-1 | Insert 36 bp sequence to pFastV8 | TCCTCCTCCGCTGCCGCGCGGCACCAGCATGAATACTGTTTCCTGTGTGAAAT |
| MF8-2 | Insert TEV site behind 36 bp sequence to pFastV8 | AACCTGTATTTTCAGGGCTCCCCTATACTAGGTTATTGGAAAAT |
| MR8-2 | Insert TEV site behind 36 bp sequence to pFastV8 | CTGAAAATACAGGTTTTCGATGATGATTCCTCCTCCGCTGCCG |
| MF9 | Insert 36 bp sequence to pFastV9 | CGCGGCAGCGGATCCGAATTCGAGCTCAAGCTTGCGGCCGCATAATGCTTAA |
| MR9 | Insert 36 bp sequence to pFastV9 | TTCGGATCCGCTGCCGCGCGGCACCAGCGCCGAGCTCGAATTCGGATCCTGG |
| MF10 | Insert 36 bp sequence to pFastV10 | CGCGGCAGCGGAGGAGGAATCATCATCCTCGAGGGATCCGAATTCAAGCTTG |
| MR10 | Insert 36 bp sequence to pFastV10 | TCCTCCTCCGCTGCCGCGCGGCACCAGATGATGATGATGATGATGCACTTTG |
| MF11 | Insert 36 bp sequence to pFastV11 | GGAGGAGGAATCATCATCCATATGCTCGAGGATCCGGCTGCT |
| MR11 | Insert 36 bp sequence to pFastV11 | GATGATGATTCCTCCTCCGCTGCCGCGCGGCACCAGGCCGCTG |
| MF12 | Insert 36 bp sequence to pFastV12 | CGCGGCAGCGGAGGAGGAATCATCATCGGTATGAAAGAAACCGCTGCTGCT |
| MR12 | Insert 36 bp sequence to pFastV12 | TCCTCCTCCGCTGCCGCGCGGCACCAGGCTACCGCGTGGCACCAGACCAGAA |
