# Supplementary material for: High-throughput FastCloning technology: A low-cost method for parallel cloning
Source: PLoS One. 2022 Sep 9;17(9):e0273873. doi: 10.1371/journal.pone.0273873 (PMC9462701; doi:10.1371/journal.pone.0273873)
Supplement: S3 Table — (DOCX) [file pone.0273873.s009.docx]

S3 Table. Primers used to test the recombination efficiencies.

| Primer | Sequence |
| --- | --- |
| High-F | GGAGGAGGAATCATCATC |
| High-R | GCTGCCGCGCGGCACCAG |
| Mqo-HF | GGAGGAGGAATCATCATCATGTCAGACCTAGCCAGAAC |
| Mqo-HR_1_ | GCTGCCGCGCGGCACCAGTCATGCGGCACCTAACTTCA |
| Mqo-HR_2_ | GCTGCCGCGCGGCACCAGTGCGGCACCTAACTTCAGCG |
| ThrS-HF | GGAGGAGGAATCATCATCATGAGCGCCCCCGCACAACC |
| ThrS-HR_1_ | GCTGCCGCGCGGCACCAGTCACTCACGACCGGCCACTT |
| ThrS-HR_2_ | GCTGCCGCGCGGCACCAGCTCACGACCGGCCACTTTCA |
| IleS-HF | GGAGGAGGAATCATCATCATGACCGATAACGCATATCC |
| IleS-HR_1_ | GCTGCCGCGCGGCACCAGTCAGGTCTTTTCGATGCTTA |
| IleS-HR_2_ | GCTGCCGCGCGGCACCAGGGTCTTTTCGATGCTTACCC |
