# Supplementary material for: High-throughput FastCloning technology: A low-cost method for parallel cloning
Source: PLoS One. 2022 Sep 9;17(9):e0273873. doi: 10.1371/journal.pone.0273873 (PMC9462701; doi:10.1371/journal.pone.0273873)
Supplement: S4 Table — (DOCX) [file pone.0273873.s010.docx]

S4 Table. Sequencing primers to high-throughput vector.

| **Vectors** | **Primers** |
| --- | --- |
| pSDB1/B2/B3/B4/B11/B12 | T7-F/T7T-R |
| pSDB5 | MBP-SF/T7T-R |
| pSDB6 | T7/MBPC-SR |
| pSDB7 | pGEX5F/pGEX3R |
| pSDB8 | pGEX6F/pGEX4R |
| pSDB9 | Upstream-F/Down1-R |
| pSDB10 | pCold-F/pCold-R |
