# Supplementary material for: High-throughput FastCloning technology: A low-cost method for parallel cloning
Source: PLoS One. 2022 Sep 9;17(9):e0273873. doi: 10.1371/journal.pone.0273873 (PMC9462701; doi:10.1371/journal.pone.0273873)
Supplement: S5 Table — (DOCX) [file pone.0273873.s011.docx]

S5 Table. The sequence of sequencing primers.

| **Primers** | **Sequence** |
| --- | --- |
| T7-F | TAATACGACTCACTATAGGG |
| T7T-R | GCTAGTTATTGCTCAGCGG |
| MBP-SF | ACAACAACCTCGGGATCG |
| MBPC-SR | ATCGCCGTTAATCCAGATTA |
| pGEX5F | GGGCTGGCAAGCCACGTTTGGTG |
| pGEX3R | CCGGGAGCTGCATGTGTCAGAGG |
| pGEX6F | TAGCTCACTCATTAGGCACC |
| pGEX4R | TTCTGAAATGAGCTGTTGAC |
| pCold-F | ACGCCATATCGCCGAAAGG |
| pCold-R | GGCAGGGATCTTAGATTCTG |
| Upstream-F | ATGCGTCCGGCGTAGA |
| Down1-R | GATTATGCGGCCGTGTACAA |
