# Supplementary material for: High-throughput FastCloning technology: A low-cost method for parallel cloning
Source: PLoS One. 2022 Sep 9;17(9):e0273873. doi: 10.1371/journal.pone.0273873 (PMC9462701; doi:10.1371/journal.pone.0273873)
Supplement: S6 Table — (DOCX) [file pone.0273873.s012.docx]

S6 Table. Comparison of molecular cloning kit at home and abroad.

| Company or method | Kit | Vectors required | Inserts required | Primers required | Incubation in Viro |
| --- | --- | --- | --- | --- | --- |
| Tsingke, China | Trelief™ SoSoo Cloning Kit | No provided  Amplify N rections | Amplify N rections | 4N | 55℃ 15min |
| Vazyme, China | relief™ SoSoo Cloning Kit | No provided  Amplify N rections | Amplify N rections | 4N | 50℃ 15min |
| Takara, Japan | In-Fusion HD Cloning Kit | No provided  Amplify N rections | Amplify N rections | 4N | 37℃ 30min |
| Genescript, China | CloneEZ® PCR Cloning Kit | No provided  Amplify N rections | Amplify N rections | 4N | 22℃ 30min + 0℃ 5min |
| NEB, USA | Gibson Assembly® Master Mix | No provided  Amplify N rections | Amplify N rections | 4N | 55℃ 15-60min |
| Thermo Fisher, USA | GeneArt Seamless Cloning | No provided  Amplify N rections | Amplify N rections | 4N | 25℃ 15-30min |
| Dogene, China | Fast Seamless Cloning | No provided  Amplify N rections | Amplify N rections | 4N | 25℃ 30min + 60℃15min |
| HTFC | Second generation high-throughput cloning | Provided | Amplify 2 rections | 3 | No need |

N represent the number of the kinds of the vector.
